# Common mental disorders and socioeconomic status in adolescents of ERICA

**DOI:** 10.11606/S1518-8787.2020054001197

**Published:** 2019-12-23

**Authors:** Isabel Batista da Silva Ribeiro, Marcia Mara Correa, Gabriela Oliveira, Nágela Valadão Cade

**Affiliations:** I Centro Universitário do Paraná. Departamento de Saúde. Curitiba, PR, Brasil; II Universidade Federal do Espírito Santo. Hospital Universitário Cassiano Antônio de Moraes. Vitória, ES, Brasil; III Universidade Federal do Espírito Santo. Programa de Pós-Graduação em Saúde Coletiva. Vitória, ES, Brasil; IV Universidade Federal do Espírito Santo. Departamento de Enfermagem. Vitória, ES, Brasil

**Keywords:** Adolescent, Mental Disorders, epidemiology, Risk Factors, Socioeconomic Factors

## Abstract

**INTRODUCTION:**

Adolescence is a stage of great social, family and emotional demands, and the literature has related common mental disorder (CMD) with poor living conditions.

**OBJECTIVE:**

To investigate the relationship between CMD and socioeconomic status in Brazilian adolescents aged 12 to 17 years.

**METHOD:**

This is a cross-sectional study with data from the Study of Cardiovascular Risk in Adolescents (ERICA – *Estudo de Riscos Cardiovasculares em Adolescentes*). The outcome was CMD and the exposure was socioeconomic status assessed by race/skin color, maternal schooling, resident/room relationship, type of school, existence of maid and bathroom at home, and work activity. For the calculation of prevalence, the survey mode was used and, in the multivariate analysis, logistic regression with p < 5%, as well as the 95% confidence interval.

**RESULTS:**

The prevalence of CMD in girls was 23.3%, and in boys, 11.1%. The variables associated with CMD in girls were age between 15 and 17 years (OR = 1.34; 1.17–1.51), studying in private school (OR = 1.13; 1.01–1.27), having a housemaid (OR = 1.15; 1.00–1.34) and, as a protective factor, unpaid work (OR = 0.64; 0.55–0.75). Boys also had a higher chance of CMD in the highest age group (OR = 1.42; 1.18–1.71) and when they had a housemaid (OR = 1.26; 1.02–1.57), whereas unpaid work decreased this chance (OR = 0.79; 0.67–0.95).

**CONCLUSION:**

Socioeconomic variables that were associated with CMD were suggestive of higher economic class, whereas unpaid work favored the mental health of adolescents, results contrary to the literature on socioeconomic status and CMD.

## INTRODUCTION

Common mental disorders (CMD) refer to two main diagnostic categories, depressive disorders and anxiety disorders, considered “common” due to their widespread prevalence in the population. These conditions affect mood and feelings, and symptoms can vary in severity and duration. The prevalence of these conditions has increased, especially in developing countries because, in addition to a growing population, more individuals reach the ages at which depression and anxiety appear^1^.

The American continent has the highest prevalence of anxiety, with Brazil showing the highest rates of these disorders, as 9.3% of the Brazilian population suffers from anxiety and 5.8% suffers depression^2^.

Despite being more prevalent during adulthood, depression also occurs in children and adolescents under 15 years of age. Regarding anxiety, the rates are similar in all age groups – perhaps lower in older adults^2^.

Studies on psychiatric epidemiology have shown a consistent association between social inequality and CMD^3^^,^^1^. The greater vulnerability of individuals in worse socioeconomic status comes from the feeling of insecurity, lack of hope, and risk of violence. On the other hand, costs with the disease worsen the economic condition^4^.

In Brazil, CMD among the adult population is becoming increasingly frequent in women, black individuals and people with “divorced” marital status or who have a bad relationship with their partner^1^. These disorders have also been associated with stressful producing events, lack of social support, precarious working conditions, unemployment, low schooling and income, limited possession of durable goods, and poor housing conditions^1^^,^^4^. The World Health Organization (WHO)^2^ reiterates this idea regarding depression as a CMD when it states that poverty, unemployment, negative life events, disruption of affective relationships, physical illnesses, and the use of alcohol and drugs increase its risk.

Few population-based articles with adolescents have been found, and part of them – both in Brazil and worldwide – emphasize the young person with some chronic disease or living condition such as pregnancy, immigration, food insecurity and their effect on mental health; in these studies, prevalence reached 43%^5^^–^^7^. In the few population-based Brazilian studies, the prevalence ranged from 28 to 30% and was higher in girls^8^^,^^9^.

Specifically addressing socioeconomic and CMD issues in young people, Brazilian^1^^,^^8^ and foreign^9^^–^^12^ studies also relate low income, low maternal schooling and health access inequities – factors that indicate worse socioeconomic status.

An English cohort study showed that the low socioeconomic status and consequent material difficulty of parents at the birth of children was associated with an increase in the early incidence of depression symptoms in adolescence, and the presence of depression symptoms in childhood and adolescence increased the chance of depression by seven times at the age of 18^10^.

In Ethiopia, 1,521 adolescents aged 17 to 21 years were evaluated considering the mechanisms by which food insecurity was associated with CMD. Low socioeconomic position, parents with few years of schooling and families headed by women were identified. For the authors, poor living conditions lead to social exclusion, stress, decreased social capital, and risk of violence for young people^11^.

Worldwide, about 20% of adolescents have mental health problems or dysfunctional behaviors, having depression as the main isolated factor that contributes to the worldwide burden of diseases in individuals aged 15 to 19 years. We thus highlight the close relationship between depression and anxiety and this stage of life. Moreover, poor mental health at earlier ages can predict mental illness in adulthood, showing the importance of knowing the mental state of young people, as well as their relationship with their economic situation^12^.

Given this context, this study aims to investigate the relationship between common mental disorders and the socioeconomic status of Brazilian adolescents aged 12 to 17 years.

## METHODS

This is a cross-sectional study using data from the *Estudo de Riscos Cardiovasculares em Adolescentes* (ERICA – Study of Cardiovascular Risk Factors in Adolescents), conducted in 2013 and 2014, which aimed to investigate the prevalence of metabolic syndrome, diabetes and cardiovascular risk factors in adolescents aged 12 to 17 years properly enrolled in public and private schools in Brazilian municipalities with more than 100,000 inhabitants.

Data were collected by trained personnel with the help of a self-administered questionnaire available in electronic devices, in which adolescents answered approximately 100 questions that comprised sociodemographic aspects, occupation, physical activity, diet, tobacco and alcohol use, sleep, morbidities, reproductive health, oral health, and mental health. Information about the school was collected directly by the researchers, and the questionnaire was also sent to parents or guardians to obtain more information on maternal schooling, and family history of cardiovascular or metabolic diseases, as well as questions related to birth and breastfeeding conditions. Anthropometric measurements, blood pressure measurement, and biochemical tests were also collected^13^.

In this study, the outcome variable was the common mental disorder, evaluated by Goldberg's General Health Questionnaire (GHQ)^14^ validated for the Brazilian population, an one-dimensional index to measure psychological morbidity. The evaluation of the factor analysis of GHQ-12 in young people aged 16 to 24 years living in Porto Alegre (RS) showed that the scale has three factors: self-esteem, depression and perceived self-efficacy; with the capacity to explain 52.7% of the total variance of responses to the GHQ^15^. A survey of Australians aged 11 to 15, showed that adolescents interpreted GHQ-12 in a similar way to adults^16^.

There are several ways to score GHQ. The binary system with cutoff point 5 was used, i.e. the presence of CMD was considered when at least 5 of the 12 items were answered with one of the last two options (“a little more than usual” or “much more than usual”). There are authors who opt for a score that favors an optimal relationship between sensitivity and specificity, using 12 or more points in a Likert system of 0 to 3 points, i.e. four or more questions with positive answers^17^. Another study with data from ERICA^18^ used a smaller cutoff point of 3 points, favoring the sensitivity of the instrument rather than specificity. In this study, the 5-point criterion was chosen due to its lower error rate for the classification of CMD and excellent validity, with 73% sensitivity, 90% specificity, and 61.2% positive predictive value^19^. The very author of the GHQ showed that the high cutoff point provides greater sensitivity (86.7%), specificity (88.9%), positive predictive value (71.2%), and ROC curve area (0.94)^21^. The score 5 was also used in young people aged 16 and 24 years from a study in India to evaluate the factors associated with CMD^20^.

Sociodemographic variables constituted exposure: age, race/color, maternal schooling, number of residents per room, number of bathrooms, presence of maid, type of school (public or private), school region (urban or rural), and work with or without remuneration. Given the large percentage of information loss about economic class (around 31%), this variable was not considered in the analyses; therefore, proxy variables about economic condition were weighted, e.g., the presence of maid and number of bathrooms^21^.

The sample used in the analyses were 74,589 students who filled the information about mental health, out of 102,237 eligible participants aged 12 to 17 years in total. Results of previous studies – including some using the ERICA population – have consistently pointed out the higher prevalence of common mental disorders among girls when compared with boys, thus justifying the presentation of the results stratified by sex.

The association between explanatory variables and CMD was evaluated using Pearson's χ^2^ test, with 0.05 significance and odds ratio with 95%CI by multinomial logistic regression. The variables that were associated with CMD with p ≤ 0.20 were entered on the multivariate analysis. The variables were adjusted and those with 0.05 statistical significance were maintained. The analyses were corrected by the complex design of the sample, using the set of SVY commands of Stata version 13.0.

This study was approved by the Research Ethics Committees (REC) of the institution of the central coordination of the study (IESC/UFRJ – process 45/2008) and the 27 research institutions participating as representatives of the states. For the participation in the study, all adolescents signed the assent form and, when required by the local REC, parents or guardians signed the informed consent form.

## RESULTS

The participants of this school-based study were 74,589 adolescents aged 12 to 17 years, of the 102,327 eligible participants of the larger study. The 27,738 losses – representing 27.1% of the total population studied – came from the lack of information about the CMD variable.

The overall prevalence of CMD, using the score of 5 or more, was 17.2% (95%CI 16.5–17.8), with a statistically significant difference between genders. The highest prevalence was observed in girls, with 23.3% (95%CI 22.3–24.4), whereas in men it was 11.1% (95%CI 10.2–11.9).

Table 1 shows the distribution of CMD prevalence, according to sex, by sociodemographic variables. The significant associations in the bivariate analysis were: being in the age group from 15 to 17 years (p < 0.001), being Asian or indigenous for the male group (p = 0.045), having monthly housemaid (p = 0.029 for women, and p = 0.036 for males), currently working in a paid or unpaid manner for women (p < 0.001), unpaid work for men (p = 0.001), and studying in urban region for women (p = 0.021).

**Table 1:** Distribution of the presence and absence of CMD according to sociodemographic variables in adolescents. ERICA, 2013–2014.

Variables		Women (n = 41,225)					Men (n = 33,364)				
		Absence of CMD (%)		Presence of CMD (%)		p	Absence of CMD (%)		Presence of CMD (%)		p
Age in years	12 to 14	79.6	(78.4–80.6)	20.4	(19.3–21.6)	< 0.001	90.7	(89.4–91.8)	9.3	(8.1–10.6)	< 0.001
	15 to 17	73.5	(71.6–75.3)	26.5	(24.7–28.4)		86.9	(85.6–88.1)	13.1	(11.8–14.4)	
Race/skin color	White	76.3	(74.6–77.8)	23.7	(22.2–25.3)	0.488	89.2	(87.5–90.6)	10.8	(9.4–12.4)	0.045
	Non-white	77.1	(75.7–78.4)	22.9	(21.6–24.2)		89.3	(88.3–90.2)	10.7	(9.8–11.7)	
	Asian or indigenous	75.0	(70.2–79.1)	25	(20.8–29.7)		83.0	(76.3–88.0)	17	(11.9–23.6)	
Maternal schooling in years	< 8	77.8	(76.3–79.2)	22.2	(20.8–23.6)	0.296	89.5	(88.2–90.6)	10.5	(9.3–11.7)	0.869
	≥ 8	76.6	(74.5–78.5)	23.4	(21.4–25.4)		89.3	(87.5–90.8)	10.6	(9.1–12.4)	
Number of residents per room in the household	< 1	76.9	(75.6–78.1)	23.1	(21.8–24.3)	0.804	88.7	(87.6–89.7)	11.3	(10.2–12.3)	0.654
	1	76.2	(73.6–78.5)	23.8	(21.4–26.3)		89.8	(87.6–91.5)	10.2	(8.4–12.3)	
	> 1	76.4	(74.6–78.1)	23.6	(21.8–25.3)		89	(87.1–91.0)	10.8	(8.9–12.9)	
Number of bathrooms in the household	None	87.7	(72.6–94.9)	12.3 23.123.8	(5.0–27.4)	0.220	83.5	(66.6–92.7)	16.5	(7.3–33.3)	0.549
	1	76.9	(75.6–78.1)		(21.8–24.4)		89.1	(88–90)	10.9	(9.9–11.9)	
	2 or more	79.2	(74.7–77.5)		(22.4–25.3)		88.8	(87.6–89.9)	11.2	(10.1–12.4)	
Monthly housemaid	No	77.2	(76.0–78.3)	22.8	(21.6–23.9)	0.029	89.5	(88.5–90.3)	10.5	(9.6–11.4)	0.036
	Yes	74.4	(71.9–76.6)	25.6	(23.3–28.0)		87.1	(84.7–89.2)	12.9	(10.8–15.2)	
Unpaid adolescent work	No	78.3	(77.1–79.3)	21.730.9	(20.6–24.2)	< 0.001	89.7	(84.9–88.2)	10.3	(9.3–11.3)	0.001
	Yes	69.1	(69.4–71.6)		(28.3–33.6)		86.7	(88.6–90.7)	13.3	(11.7–15.0)	
Paid adolescent work	No	77.1	(75.9–78.1)	22.9	(21.8–24.1)	< 0.001	89	(88–89.9)	11	(10.0–12.0)	0.739
	Yes	67.9	(62.2–73.0)	32.1	(26.9–37.7)		88.4	(84.3–91.4)	11.6	(8.5–15.6)	
Type of school	Public	77.0	(75.2–78.1)	23	(21.8–24.2)	0.062	88.9	(87.9–89.7)	11.1	(11.1–12.1)	0.839
	Private	75.2	(73.6–76.6)	24.8	(23.3–26.3)		89.1	(87.6–90.4)	10.9	(9.6–12.4)	
School region	Urban	76.4	(75.3–77.4)	23.6	(22.6–24.6)	0.021	88.9	(87.9–89.7)	11.1	(10.2–12.0)	0.325
	Rural	83.7	(77.7–88.2)	16.3	(11.7–22.3)		90	(87.8–91.8)	10	(8.2–12.1)	

CMD: common mental disorders

Table 2 shows the association between the independent variables investigated in relation to the outcome. All with p < 0.20 were considered possible confounding factors. In the adjusted multivariate analysis, the association between CMD and the variables analyzed remains even with control for possible confounding factors, except for the variables paid work and school region, both for women. Girls had a higher chance of CMD in the highest age group (OR = 1.34; 95%CI 1.17–1.51), with the presence of a monthly housemaid at home (OR = 1.15; 95%CI 1.00–1.34), and private school education (OR = 1.13; 95%CI 1.01–1.27), whereas unpaid work reduced this chance by 36% (OR = 0.64; 95%CI 0.55–0.75). For boys, the results were similar to those found in girls, except for studying in a private school.

**Table 2:** Crude and adjusted analysis of common mental disorders according to sociodemographic variables. ERICA, 2013–2014.

Variables		Women (n = 41,225)				Men (n = 33,364)			
		Crude	p	Adjusted	p	Crude	p	Adjusted	p
Age in years	12 to 14	1.0	0.000	1.0	0.000	1.0	0.000	1.0	0.000
	15 to 17	1.40 (1.25–1.57)		1.34 (1.17–1.51)		1.46 (1.21–1.77)		1.42 (1.87–1.71)	
Race/skin color	White	1.0	0.591			1.0	0.350		
	Non-white	0.95 (0.86–1.06)				0.99 (0.82–1.19)			
	Asian or indigenous	1.07 (0.83–1.38)				1.68 (1.11–2.56)			
Maternal schooling in years	< 8	1.0	0.296			1.0	0.739		
	≥ 8	1.07 (0.94–1.22)				1.01 (0.82–1.26)			
Number of residents per room in the household	< 1	1.0	0.588			1.0	0.565		
	1	1.04 (0.91–1.18)				0.89 (0.70–1.13)			
	> 1	1.02 (0.91–1.15)				0.95 (0.75–1.20			
Number of bathrooms in the household	None	1.0	0.301			1.0	0.870		
	1	2.12 (0.81–5.59)				0.61 (0.24–1.55)			
	2 or more	2.21 (0.84–5.87)				0.63 (0.25–1.62)			
Monthly housemaid	No	1.0	0.030	1.0	0.049	1.0	0.037	1.0	0.031
	Yes	1.17 (1.01–1.34)		1.15 (1.00–1.34)		1.26 (1.01–1.56)		1.26 (1.02–1.57)	
Unpaid adolescent work	No	1.0	0.000	1.0	0.000	1.0	0.001	1.0	0.011
	Yes	0.62 (0.54–0.71)		0.64 (0.55–0.75)		0.74 (0.62–0.89)		0.79 (0.67–0.95)	
Paid adolescent work	No	1.0	0.001	1.0	0.988	1.0	0.565		
	Yes	1.58 (1.21–2.08)		1.00 (0.75–1.32)		1.06 (0.73–1.56)			
Type of school	Public	1.0	0.062	1.0	0.031	1.0	0.840		
	Private	1.10 (0.99–1.22)		1.13(1.01–1.27)		0.98 (0.82–1.17)			
School region	Urban	1.0	0.023	1.0	0.052	1.0	0.325		
	Rural	0.63 (0.42–0.93)		0.69 (0.48–1.00)		0.88 (0.69–1.12)			

## DISCUSSION

This study provided an estimate of CMD prevalence, using the GHQ with cutoff point 5 and its association with exposures related to the socioeconomic status of Brazilian adolescents. The overall prevalence and prevalence by gender was lower than those found in Brazilian studies with adolescents in school environments^7^^,^^8^^,^^22^. However, different assessment instruments have been used, as well as ways to punctuate the GHQ, thus making comparisons difficult.

The study by Lopes^22^, also conducted with ERICA data, showed higher prevalence because it used 3 points as the cutoff point, corresponding to the sensitivity of the instrument, whereas this study considered 5 points, which is compatible with the specificity of GHQ^20^. CMD prevalence was thus expected to be lower in this study. In Brazilian studies in which adolescents had some specific condition, CMD prevalence was also higher when compared with this study, showing that adolescents from health services undergoing treatment for chronic diseases presented worse mental health^5^^,^^6^.

Those aged between 15 and 17 years presented higher CMD prevalence than younger individuals, because this is a stage of life of greater burden of anxiety derived from the search for identity, professional choice, and insertion in the adult world^23^. As in other studies^7^^,^^8^, CMD prevalence among girls was higher. The literature reports that depression and anxiety prevalence is two to three times higher in girls, and that they have better self-perception of their self and health, expressing their symptoms more easily and presenting the behavior to seek health services more frequently^1^. In girls, the existence of a monthly housemaid and studying in a private school were risk factors for worse mental health, whereas unpaid work was a protective factor. The results for boys were similar, except for studying in a private school.

A survey conducted in Rio de Janeiro with high school adolescents in public schools showed that, despite the difficulty in reconciling work with school faced by adolescents, the work experience allows more independence, conquest of autonomy, and outline life projects^23^.

Domestic activity the unpaid work reported the most by the adolescents of ERICA. In a study conducted in Portugal, young people who collaborated with this type of activity had better school performance^24^.

Studies have also shown that extracurricular activities have been associated with beneficial effects on the development of adolescents regarding academic performance, self-esteem, and motivational and cognitive aspects, in contrast to the unstructured free time. The emotional adaptation of adolescents is negatively affected when most of their free time is not structured. Those who participate in extracurricular activities have a protective effect in relation to depression, delinquency, and risky behaviors such as alcohol, smoking and drugs^24^^,^^25^. Authors have argued that, when there is no exploitation, work activities can be positive for adolescents by enabling the expression of skills, creativity and favoring the professional development of young individuals^25^.

Another variable that was associated with CMD in this study was to study in a private school, suggesting that better living conditions favors a worse mental health status. A higher level of stress has already been observed in private school students (77% of them) when compared with public school students (51%), with symptoms of the most severe phase being more frequent in students of higher classes; the authors of the study argue that these young individuals are under more pressure from family members and teachers for better academic performance so they achieve a good future insertion in undergraduate courses^26^.

Adolescence is a period of frailty for some young people and may potentiate the emergence of stressful events and crises resulting from physical, psychological, social, and cultural changes. The demand for choosing a profession is a characteristic of this stage of life, and with this comes a period with more school activities and the expectation of family members – and the very self-expectation – for results that enable the fulfillment of a given professional future.

There are studies with adolescent populations that evaluated CMD and found an association with poor socioeconomic status. One study, conducted with adolescents from Ethiopia, observed that the probability increased when participants faced food insecurity, lived in families with low schooling and headed by women, and lived in urban centers.

Using an index of wealth, a reduction in CMD was observed in young people as household wealth increased, showing that social inequity contributes to poor mental health^11^. A cohort study followed young subjects aged 10 to 18 years regarding the onset of depressive symptoms and their relationship with the previous socioeconomic position of the family, evaluated by the occupation of parents, maternal schooling in years, and standard of living (material difficulties, owning a house, and access to car).

There was an association between depression at 18 years of age and exposure to financial problems and material difficulties^10^. An evaluation of the general population starting from 15 years of age found an association between CMD and low income and illiteracy, understanding that socioeconomic factors affect mental health in all stages of life^17^. In Brazil, adolescents with mothers with fewer schooling years had a higher CMD prevalence than the children of mothers with over eight years of schooling^7^^,^^8^.

Studies with adolescents have used maternal schooling more often than socioeconomic status. In this study, this information was given by the adolescent via a self-administered instrument, and it showed the incompleteness of data regarding the schooling of the head of the family; thus, making the economic classification impossible. We thus propose that maternal schooling may have faced information bias, and that the use of other proxy variables may have provided a greater understanding of the questions.

The variables used to evaluate the living and socioeconomic condition in this study were proxy ones such as race/skin color, maternal schooling, number of residents per room, number of bathrooms, presence of a monthly housemaid, type of school, school region and occupational activity of the young person. Considering the results, we must question if such measurements express the socioeconomic status of the adolescents.

Ewerling et al.^21^ evaluated the ownership of goods by the Brazilian economic indicator, also common to the Brazilian Criteria of *Associação Brasileira de Empresas de Pesquisa*, both created as proxy instruments to express the wealth of households. The study used data from biannual surveys conducted in Pelotas (RS) between 2002 and 2014, and only in 2012 there were adolescents. Our study used three of the variables in isolation – monthly housemaid, number of bathrooms, and resident/room relationship – as markers of rich households21^21^.

The evaluation of the relationship between poverty and mental health in low- and middle-income countries has shown conflicting results, possibly due to the variables selected to assess socioeconomic status, which are not based on a single definition of poverty. In studies with socioeconomic variables such as education, income and others, it is common to see a positive association in the bivariate analysis that weakens, disappears or becomes negative in the adjusted analysis^27^. This also occurred in this study since maternal schooling only showed a difference in the crude analysis, which was not maintained in the adjusted analysis.

Measurement problems using screening instruments such as GHQ, the way to measure the variable ‘poverty,' and population factors may explain the variability of the association found in studies, as well as the factors that mediate the relationship between poverty and CMD. On the other hand, the knowledge that poverty causes CMD does not exclude the reverse, thus requiring cohort studies. There are specific dimensions of poverty that lead to CMD, and the paths to be traveled are complex because socioeconomic status is a social and multidimensional construction^10^.

As limitations of this study, we note that all instruments used with children and adolescents are susceptible to information biases. GHQ-12 is a widely validated instrument in adults, but further studies with adolescents are needed. However, we found studies on the factorial analysis of the GHQ-12 applied in young Brazilians^14^ and Australians^16^, and a longitudinal study that evaluated the cultural sensitivity of the instrument, showing that it is culturally sensitive to various ethnic groups in England^28^. This instrument also present different forms of scoring, which makes it difficult to compare our results with those of other studies. However, GHQ has been the instrument of choice in epidemiological studies on the subject, whether with adults or young people.

Socioeconomic variables that were positively associated with CMD in adolescents – e.g., having a housemaid and studying in private schools – suggest the belonging to higher economic classes, and that this condition may create an environment that causes mood changes. Unpaid work favored the mental health of adolescents and represents a type of activity that limits idleness and exposure to risk environments. The results were distinct from the literature on socioeconomic status and CMD; we recommend that further studies are conducted with Brazilian adolescents to investigate external validation.
