# Supplementary material for: Common mental disorders and socioeconomic status in adolescents of ERICA
Source: Rev Saude Publica. 2019 Dec 23;54:04. doi: 10.11606/S1518-8787.2020054001197 (PMC9586442; doi:10.11606/S1518-8787.2020054001197)
Supplement: Translation

# Translation: Transtorno mental comum e condição socioeconômica em adolescentes do Erica

**Authors:** Isabel Batista da Silva Ribeiro, Marcia Mara Correa, Gabriela Oliveira, Nágela Valadão Cade

**Affiliations:** I Centro Universitário do Paraná. Departamento de Saúde. Curitiba, PR, Brasil; II Universidade Federal do Espírito Santo. Hospital Universitário Cassiano Antônio de Moraes. Vitória, ES, Brasil; III Universidade Federal do Espírito Santo. Programa de Pós-Graduação em Saúde Coletiva. Vitória, ES, Brasil; IV Universidade Federal do Espírito Santo. Departamento de Enfermagem. Vitória, ES, Brasil

**Keywords:** Adolescente, Transtornos Mentais, epidemiologia, Fatores de Risco, Fatores Socioeconômicos

## Abstract

**INTRODUÇÃO:**

A adolescência é uma fase de grande demanda social, familiar e emocional, e a literatura tem relacionado o transtorno mental comum (TMC) com piores condições de vida.

**OBJETIVO:**

Investigar a relação entre TMC e a condição socioeconômica em adolescentes brasileiros de 12 a 17 anos.

**MÉTODO:**

Estudo seccional com os dados do Estudo de Riscos Cardiovasculares em Adolescentes (Erica). O desfecho foi o TMC e a exposição foi a condição socioeconômica avaliada por raça/cor, escolaridade materna, relação morador/cômodo, tipo de escola, existência de empregada e banheiro no domicílio e atividade laboral. Para o cálculo das prevalências, foi utilizado o modo *survey* e, na análise multivariada, a regressão logística com p < 5%, assim como o intervalo de confiança de 95%.

**RESULTADOS:**

A prevalência de TMC em meninas foi 23,3% e em meninos, 11,1%. As variáveis associadas ao TMC nas meninas foram ter idade entre 15 e 17 anos (OR = 1,34; 1,17–1,51), estudar em escola privada (OR = 1,13; 1,01–1,27), ter empregada doméstica (OR = 1,15; 1,00–1,34) e, como fator de proteção, o trabalho não remunerado (OR = 0,64; 0,55–0,75). Os meninos também apresentaram maior chance de TMC na faixa etária mais alta (OR = 1,42; 1,18–1,71) e quando tinham empregada (OR = 1,26; 1,02–1,57), enquanto o trabalho não remunerado diminuiu essa chance (OR = 0,79; 0,67–0,95).

**CONCLUSÃO:**

As variáveis socioeconômicas que estiveram associadas ao TMC foram sugestivas de classe econômica mais elevada, enquanto o trabalho não remunerado favoreceu a saúde mental dos adolescentes, resultados contrários à literatura sobre condição socioeconômica e TMC.

## INTRODUÇÃO

Os transtornos mentais comuns (TMC) referem-se a duas categorias diagnósticas principais, as depressivas e as de ansiedade, consideradas “comuns” por serem muito prevalentes na população. No entanto, elas têm impacto no humor e nos sentimentos, e os sintomas podem variar em gravidade e duração. A prevalência desses quadros tem aumentado, principalmente em países de baixa renda, pois, além de a população estar crescendo, mais indivíduos chegam a idades nas quais a depressão e a ansiedade aparecem^1^.

De forma isolada, o continente americano detém a maior prevalência de ansiedade, e o Brasil apresenta as maiores taxas dessas condições entre os países das Américas, com 9,3% da população brasileira com ansiedade e 5,8% com depressão^2^.

De forma geral, apesar de ser mais prevalente na fase adulta, a depressão também ocorre em crianças e adolescentes com menos de 15 anos. Quanto à ansiedade, as taxas são semelhantes em todos os grupos etários – talvez menores em idosos^2^.

Estudos sobre epidemiologia psiquiátrica têm mostrado uma associação consistente entre a desigualdade social e os TMC^3^^,^^1^. A maior vulnerabilidade dos indivíduos em pior condição socioeconômica advém da sensação de insegurança, falta de esperança e risco de violência. Por outro lado, os custos com a doença pioram a situação econômica^4^.

No Brasil, na população adulta, os TMC têm se mostrado mais frequentes em mulheres, negros e em pessoas com estado civil “separadas” ou que têm um relacionamento considerado ruim com seu companheiro^1^. Também tem sido associado a eventos produtores de estresse, inexistência de apoio social, condições de trabalho precárias, desemprego, baixa escolaridade e renda, pequena posse de bens duráveis e más condições de moradia^1^^,^^4^. A Organização Mundial da Saúde (OMS)^2^ reitera essa ideia no que diz respeito à depressão enquanto TMC quando afirma que condições como pobreza, desemprego, eventos negativos de vida, rompimentos afetivos, doenças físicas e uso de álcool e drogas aumentam o seu risco.

Poucos artigos de base populacional com adolescentes foram encontrados, e parte deles, tanto no Brasil como no mundo, dão ênfase ao jovem com alguma doença crônica ou condição de vida como gravidez, imigração, insegurança alimentar e o efeito delas sobre a saúde mental; nesses estudos a prevalência alcançou 43%^5^^–^^7^. Nos poucos trabalhos brasileiros de base populacional, a prevalência variou entre 28 e 30% e foi mais elevada em meninas^8^^,^^9^.

Especificamente abordando as questões socioeconômicas e TMC em jovens, estudos nacionais^1^^,^^8^ e internacionais^9^^–^^12^ também o relacionam à baixa renda, baixa escolaridade da mãe e iniquidades de acesso à saúde, o que indica pior condição socioeconômica.

Um estudo de coorte inglês evidenciou que a baixa condição socioeconômica e consequente dificuldade material dos pais no nascimento dos filhos foi associada ao aumento da incidência precoce de sintomas depressivos na adolescência, e a presença de sintomas depressivos na infância e adolescência aumentaram em sete vezes a chance de depressão aos 18 anos^10^.

Na Etiópia, 1.521 adolescentes de 17 a 21 anos foram avaliados quanto aos mecanismos pelos quais a insegurança alimentar associava-se aos TMC. Identificaram-se baixa posição socioeconômica, pais com poucos anos de estudo e famílias chefiadas por mulheres. Para os autores, piores condições de vida proporcionam exclusão social, estresse, diminuição do capital social e risco de violência para os jovens^11^.

Em todo o mundo, cerca de 20% dos adolescentes têm problemas de saúde mental ou comportamentos disfuncionais, sendo a depressão o principal fator isolado que contribui para a carga mundial de doenças em indivíduos entre 15 e 19 anos. Destaca-se que há uma estreita relação entre depressão e ansiedade nessa fase da vida. Além disso, uma pior saúde mental em idades mais precoces pode predizer adoecimento mental na fase adulta, o que demostra a importância de conhecer o estado mental dos jovens, bem como sua relação com a situação econômica^12^.

Nessa premissa, este estudo objetiva investigar a relação entre transtorno mental comum e a condição socioeconômica de adolescentes brasileiros de 12 a 17 anos.

## MÉTODOS

Trata-se de estudo transversal utilizando dados do Estudo de Riscos Cardiovasculares em Adolescentes (Erica), conduzido nos anos 2013 e 2014, que teve por objetivo conhecer a prevalência de síndrome metabólica, diabetes e fatores de risco cardiovascular em adolescentes de 12 a 17 anos devidamente matriculados em escolas públicas e privadas de municípios brasileiros com mais de 100 mil habitantes.

Os dados foram coletados por pessoal treinado com o auxílio de questionário autopreenchível disponível em dispositivos eletrônicos, no qual os adolescentes respondiam aproximadamente 100 questões que compreendiam aspectos sociodemográficos, ocupação, atividade física, alimentação, uso de tabaco e bebidas alcoólicas, sono, morbidades, saúde reprodutiva, saúde bucal e saúde mental.. Informações referentes à escola foram coletadas diretamente pelos pesquisadores, sendo também enviado aos pais ou responsáveis o questionário com o intuito de obter maiores informações sobre escolaridade materna e histórico familiar de doenças cardiovasculares e metabólicas, assim como questões relativas às condições de nascimento e amamentação. Medidas antropométricas, aferição de pressão arterial e exames bioquímicos também foram colhidos^13^.

Neste estudo a variável desfecho foi o transtorno mental comum, avaliado pelo Questionário Geral de Saúde (GHQ) de Goldberg^14^ validado para a população brasileira, um índice unidimensional para mensurar a morbidade psicológica. A avaliação da análise fatorial do GHQ-12 em jovens de 16 a 24 anos residentes em Porto Alegre (RS) mostrou que a escala tem três fatores, autoestima, depressão e autoeficácia percebida, com capacidade para explicar 52,7% da variância total das respostas ao GHQ^15^. Em pesquisa com australianos de 11 a 15 anos, foi evidenciado que os adolescentes interpretaram o GHQ-12 de maneira semelhante aos adultos^16^.

Há várias maneiras de pontuar o GHQ. Foi utilizado o sistema binário com o ponto de corte 5, ou seja, considerou-se presença de TMC quando pelo menos 5 dos 12 itens foram respondidos com uma das duas últimas opções (“um pouco mais que de costume” ou “muito mais que de costume”). Há autores que optam por um escore que privilegie uma ótima relação entre sensibilidade e especificidade, utilizando 12 ou mais pontos em um sistema Likert de 0 a 3 pontos, ou seja, quatro ou mais questões com respostas positivas^17^. Já outro estudo com dados do Erica^18^ utilizou um ponto de corte menor, de 3 pontos, privilegiando a sensibilidade do instrumento em vez da especificidade. Neste estudo optou-se pelo critério de 5 pontos devido a sua menor taxa de erro de classificação do TMC e ótima validade, com 73% de sensibilidade, 90% de especificidade e 61,2% de valor preditivo positivo^19^. O próprio autor do GHQ demonstrou que o ponto de corte elevado proporciona maior sensibilidade (86,7%), especificidade (88,9%), valor preditivo positivo (71,2%) e área de curva ROC (0,94)^21^. O escore 5 foi utilizado também em jovens entre 16 e 24 anos de um estudo da Índia para avaliar os fatores associados ao TMC^20^.

Variáveis sociodemográficas constituíram a exposição: idade, raça/cor, escolaridade da mãe, relação morador/cômodo, quantidade de banheiros, presença de empregada doméstica, tipo de escola (pública ou privada), região da escola (urbana ou rural) e trabalho com ou sem remuneração. Devido ao grande percentual de perda de informação sobre classe econômica (em torno de 31%), essa variável não foi considerada nas análises; por isso, variáveis *proxy* da condição econômica, como presença de empregada doméstica e quantidade de banheiros, foram ponderadas^21^.

Para as análises, foi considerada uma amostra de 74.589 escolares, que preencheram as informações relativas a saúde mental, de um total de 102.327 elegíveis na faixa etária de 12 a 17 anos. Resultados de estudos prévios, inclusive utilizando a população do Erica, têm apontado de forma consistente prevalências de transtornos mentais comuns mais elevadas entre meninas quando comparadas aos meninos, justificando assim a apresentação dos resultados estratificados por sexo.

Para avaliação da associação entre as variáveis explicativas e TMC foi utilizado o teste do χ^2^ de Pearson, com significância de 0,05 e o *odds ratio* com IC95% mediante regressão logística multinominal. As variáveis que foram associadas a TMC com p ≤ 0,20 entraram na análise multivariável. Foi realizado o ajuste das variáveis e mantida aquelas com significância estatística de 0,05. As análises foram corrigidas pelo delineamento complexo da amostra, utilizando-se o conjunto de comandos SVY do Stata versão 13.0.

O estudo foi aprovado pelos comitês de ética em pesquisa (CEP) da instituição da coordenação central do estudo (IESC/UFRJ – processo 45/2008) e das vinte e sete instituições de pesquisa participantes representantes dos estados. Para participação do estudo o adolescente assinava o termo de assentimento e, quando exigido pelo CEP local, os pais ou responsáveis assinavam o termo de consentimento livre e esclarecido

## RESULTADOS

Participaram deste estudo de base escolar 74.589 adolescentes de 12 a 17 anos dos 102.327 participantes elegíveis ao estudo maior. As 27.738 perdas, representando 27,1% da população total estudada, advieram da falta de informações sobre a variável TMC.

A prevalência geral de TMC, utilizando o escore de 5 ou mais, foi de 17,2% (IC95% 16,5–17,8), com diferença estatisticamente significante entre os sexos. A maior prevalência foi observada nas meninas, com 23,3% (IC95% 22,3–24,4), enquanto no sexo masculino o índice foi 11,1% (IC95% 10,2–11,9).

A distribuição da prevalência dos TMC, segundo sexo, por variáveis sociodemográficas, está apresentada na Tabela 1. As associações significativas na análise bivariada foram: estar na faixa etária de 15 a 17 anos (p < 0,001), ser da raça/cor asiática ou indígena para o grupo do sexo masculino (p = 0,045), ter empregada doméstica mensalista (p = 0,029 para o sexo feminino e p = 0,036 para o masculino), estar trabalhando atualmente de forma remunerada ou não remunerada para as adolescentes do sexo feminino (p < 0,001), estar trabalhando de forma não remunerada para adolescentes do sexo masculino (p = 0,001) e estudar em região urbana para as meninas (p = 0,021).

**Tabela 1:** Distribuição da presença e ausência de TMC segundo variáveis sociodemográficas em adolescentes. Erica, 2013–2014.

Variáveis		Sexo feminino (n = 41.225)					Sexo masculino (n = 33.364)				
		Ausência TMC (%)		Presença TMC (%)		p	Ausência TMC (%)		Presença TMC (%)		p
Idade em anos	12 a 14	79,6	(78,4–80,6)	20,4	(19,3–21,6)	< 0,001	90,7	(89,4–91,8)	9,3	(8,1–10,6)	< 0,001
	15 a 17	73,5	(71,6–75,3)	26,5	(24,7–28,4)		86,9	(85,6–88,1)	13,1	(11,8–14,4)	
Raça/cor	Branca	76,3	(74,6–77,8)	23,7	(22,2–25,3)	0,488	89,2	(87,5–90,6)	10,8	(9,4–12,4)	0,045
	Não branca	77,1	(75,7–78,4)	22,9	(21,6–24,2)		89,3	(88,3–90,2)	10,7	(9,8–11,7)	
	Asiática ou indígena	75,0	(70,2–79,1)	25	(20,8–29,7)		83,0	(76,3–88,0)	17	(11,9–23,6)	
Anos de estudo da mãe	< 8	77,8	(76,3–79,2)	22,2	(20,8–23,6)	0,296	89,5	(88,2–90,6)	10,5	(9,3–11,7)	0,869
	≥ 8	76,6	(74,5–78,5)	23,4	(21,4–25,4)		89,3	(87,5–90,8)	10,6	(9,1–12,4)	
Número de moradores/cômodo no domicílio	< 1	76,9	(75,6–78,1)	23,1	(21,8–24,3)	0,804	88,7	(87,6–89,7)	11,3	(10,2–12,3)	0,654
	1	76,2	(73,6–78,5)	23,8	(21,4–26,3)		89,8	(87,6–91,5)	10,2	(8,4–12,3)	
	> 1	76,4	(74,6–78,1)	23,6	(21,8–25,3)		89	(87,1–91,0)	10,8	(8,9–12,9)	
Número de banheiros no domicílio	Nenhum	87,7	(72,6–94,9)	12,3	(5,0–27,4)	0,220	83,5	(66,6–92,7)	16,5	(7,3–33,3)	0,549
	1	76,9	(75,6–78,1)		(21,8–24,4)		89,1	(88–90)	10,9	(9,9–11,9)	
	2 ou mais	79,2	(74,7–77,5)		(22,4–25,3)		88,8	(87,6–89,9)	11,2	(10,1–12,4)	
Empregada doméstica mensalista	Não	77,2	(76,0–78,3)	22,8	(21,6–23,9)	0,029	89,5	(88,5–90,3)	10,5	(9,6–11,4)	0,036
	Sim	74,4	(71,9–76,6)	25,6	(23,3–28,0)		87,1	(84,7–89,2)	12,9	(10,8–15,2)	
Trabalho adolescente não remunerado	Não	78,3	(77,1–79,3)	21,730,9	(20,6–24,2)	< 0,001	89,7	(84,9–88,2)	10,3	(9,3–11,3)	0,001
	Sim	69,1	(69,4–71,6)		(28,3–33,6)		86,7	(88,6–90,7)	13,3	(11,7–15,0)	
Trabalho adolescente remunerado	Não	77,1	(75,9–78,1)	22,9	(21,8–24,1)	< 0,001	89	(88–89,9)	11	(10,0–12,0)	0,739
	Sim	67,9	(62,2–73,0)	32,1	(26,9–37,7)		88,4	(84,3–91,4)	11,6	(8,5–15,6)	
Tipo de escola	Pública	77,0	(75,2–78,1)	23	(21,8–24,2)	0,062	88,9	(87,9–89,7)	11,1	(11,1–12,1)	0,839
	Privada	75,2	(73,6–76,6)	24,8	(23,3–26,3)		89,1	(87,6–90,4)	10,9	(9,6–12,4)	
Região da escola	Urbana	76,4	(75,3–77,4)	23,6	(22,6–24,6)	0,021	88,9	(87,9–89,7)	11,1	(10,2–12,0)	0,325
	Rural	83,7	(77,7–88,2)	16,3	(11,7–22,3)		90	(87,8–91,8)	10	(8,2–12,1)	

TMC: transtornos mentais comuns

Na Tabela 2, pode-se observar a associação entre as variáveis independentes investigadas em relação ao desfecho. Todas com p < 0,20 foram consideradas possíveis fatores de confusão. Nota-se que, na análise multivariada ajustada, a associação entre TMC e as variáveis analisadas permanece mesmo com controle para possíveis fatores de confusão, exceto para as variáveis trabalho remunerado e região da escola, ambas para o sexo feminino. Observa-se que as meninas tiveram maior chance de TMC na faixa etária mais alta (OR = 1,34; IC95% 1,17–1,51), com presença de empregada doméstica mensalista no domicílio (OR = 1,15; IC95% 1,00–1,34) e educação em escola privada (OR = 1,13; IC95% 1,01–1,27), enquanto trabalhar de forma não remunerada reduziu essa chance em 36% (OR = 0,64; IC% 0,55–0,75). Para os meninos, os resultados foram semelhantes aos encontrados nas meninas, exceto para estudar em escola privada.

**Tabela 2:** Análise bruta e ajustada dos transtornos mentais comuns, segundo variáveis sociodemográficas. Erica, 2013–2014

Variáveis		Sexo feminino (n = 41.225)				Sexo masculino (n = 33.364)			
		Bruta	p	Ajustada	p	Bruta	p	Ajustada	p
Idade em anos	12 a 14	1,0	0,000	1,0	0,000	1,0	0,000	1,0	0,000
	15 a 17	1,40 (1,25–1,57)		1,34 (1,17–1,51)		1,46 (1,21–1,77)		1,42 (1,87–1,71)	
Raça/cor	Branca	1,0	0,591			1,0	0,350		
	Não branca	0,95 (0,86–1,06)				0,99 (0,82–1,19)			
	Asiática ou indígena	1,07 (0,83–1,38)				1,68 (1,11–2,56)			
Anos de estudo da mãe	< 8	1,0	0,296			1,0	0,739		
	≥ 8	1,07 (0,94–1,22)				1,01 (0,82–1,26)			
Número de moradores/cômodo no domicílio	< 1	1,0	0,588			1,0	0,565		
	1	1,04 (0,91–1,18)				0,89 (0,70–1,13)			
	> 1	1,02 (0,91–1,15)				0,95 (0,75–1,20			
Número de banheiros no domicílio	Nenhum	1,0	0,301			1,0	0,870		
	1	2,12 (0,81–5,59)				0,61 (0,24–1,55)			
	2 ou mais	2,21 (0,84–5,87)				0,63 (0,25–1,62)			
Empregada doméstica mensalista	Não	1,0	0,030	1,0	0,049	1,0	0,037	1,0	0,031
	Sim	1,17 (1,01–1,34)		1,15 (1,00–1,34)		1,26 (1,01–1,56)		1,26 (1,02–1,57)	
Trabalho adolescente não remunerado	Não	1,0	0,000	1,0	0,000	1,0	0,001	1,0	0,011
	Sim	0,62 (0,54–0,71)		0,64 (0,55–0,75)		0,74 (0,62–0,89)		0,79 (0,67–0,95)	
Trabalho adolescente remunerado	Não	1,0	0,001	1,0	0,988	1,0	0,565		
	Sim	1,58 (1,21–2,08)		1,00 (0,75–1,32)		1,06 (0,73–1,56)			
Tipo de escola	Pública	1,0	0,062	1,0	0,031	1,0	0,840		
	Privada	1,10 (0,99–1,22)		1,13(1,01–1,27)		0,98 (0,82–1,17)			
Região da escola	Urbana	1,0	0,023	1,0	0,052	1,0	0,325		
	Rural	0,63 (0,42–0,93)		0,69 (0,48–1,00)		0,88 (0,69–1,12)			

## DISCUSSÃO

Este estudo forneceu uma estimativa da prevalência de TMC, utilizando o GHQ com ponto de corte 5 e sua associação com exposições relacionadas à condição socioeconômica de adolescentes brasileiros. A prevalência geral e por sexo foi menor do que aquelas apresentadas em pesquisas nacionais com adolescentes em ambiente escolar^7^^,^^8^^,^^22^. Todavia, diferentes instrumentos avaliativos têm sido utilizados, bem como formas de pontuar o GHQ, o que dificulta a comparação das prevalências.

O estudo de Lopes^22^, realizado também com os dados do Erica, mostrou prevalências mais elevadas, pois utilizou como ponto de corte o valor de 3 pontos, o que corresponde à sensibilidade do instrumento, enquanto este trabalho considerou o valor de 5, compatível com a especificidade do GHQ^20^. Portanto, esperava-se que a prevalência de TMC fosse menor neste estudo. Em trabalhos nacionais nos quais os adolescentes apresentavam alguma condição específica, as prevalências de TMC também se mostraram mais elevadas em relação a este estudo, evidenciando que adolescentes advindos dos serviços de saúde em tratamento de doenças crônicas apresentavam pior saúde mental^5^^,^^6^.

Aqueles com idade entre 15 e 17 anos apresentaram maior prevalência de TMC que os mais jovens, por ser uma fase de maior carga de ansiedade devido à busca pela identidade, escolha profissional e inserção no mundo adulto^23^. Como em outros estudos^7^^,^^8^, as meninas apresentaram maior prevalência de TMC. A literatura relata que elas têm duas a três vezes mais ansiedade e depressão do que os meninos e uma melhor autopercepção de si e de sua saúde, expressam mais facilmente seus sintomas e apresentam com mais frequência o comportamento de busca por serviços de saúde^1^. Em meninas, a existência de empregada doméstica mensalista no domicílio e o estudo em escola privada foram condições de risco para pior saúde mental, enquanto o trabalho não remunerado foi fator de proteção. Os resultados dos meninos foram semelhantes, exceto para estudar em escola privada.

Um levantamento realizado no Rio de Janeiro com adolescentes do ensino médio em escolas públicas mostrou que, mesmo sendo difícil para o jovem conciliar o trabalho com a escola, a experiência laboral possibilita mais independência, conquista da autonomia e delineamento de projetos de vida^23^.

O trabalho não remunerado mais relatado pelos adolescentes do Erica foi a atividade doméstica. Em um estudo realizado em Portugal, os jovens que colaboraram com esse tipo de atividade apresentaram melhor rendimento escolar^24^.

Estudos também evidenciaram que atividades extracurriculares têm sido associadas a efeitos benéficos no desenvolvimento dos adolescentes, no que diz respeito ao desempenho acadêmico, à autoestima e aos aspectos motivacionais e cognitivos, em contraste com o tempo livre não estruturado. Verificou-se efeito negativo para a adaptação emocional dos adolescentes quando a maior parte do seu tempo livre não é estruturado. Os que participam de atividades extracurriculares apresentam efeito protetor em relação a depressão, delinquência e comportamentos de risco como álcool, fumo e drogas^24^^,^^25^. Autores têm defendido que, quando não há exploração, a atividade laboral pode ser positiva para os adolescentes por possibilitar a expressão de habilidades, criatividade e favorecimento do desenvolvimento profissional do jovem^25^.

Outra variável que esteve associada ao TMC neste estudo, sugerindo que a melhor condição de vida favorece uma pior saúde mental, foi estudar em instituição privada. Já foi observado um maior nível de estresse nos alunos de escola privada (77% deles) em relação aos alunos de escola pública (51%), com sintomas da fase mais grave sendo mais frequentes nos estudantes de classes mais elevadas; os autores do trabalho argumentam que esses jovens são mais cobrados por familiares e professores para obter um melhor desempenho acadêmico, de forma que consigam uma boa inserção futura em cursos de graduação^26^.

A adolescência é um período de fragilidade para alguns jovens e pode potencializar o surgimento de eventos estressores e crises decorrentes de mudanças físicas, psicológicas, sociais e culturais. É característica dessa fase a demanda pela escolha de uma profissão, e com isso advém um período com mais atividades escolares e expectativa dos familiares ou própria quanto a resultados que viabilizem a concretização de um determinado futuro profissional.

Há trabalhos com a população de adolescentes que avaliaram TMC e encontraram associação com uma pior condição socioeconômica. Um deles, com adolescentes da Etiópia, observou que a probabilidade aumentava quando eles apresentavam insegurança alimentar, viviam em famílias de baixa escolaridade e chefiadas por mulheres e residiam em centros urbanos. Mediante um índex de riqueza, foi constatada redução de TMC nos jovens à medida que a riqueza do agregado familiar aumentava, evidenciando que a iniquidade social contribui para uma pior saúde mental^11^. Um estudo de coorte acompanhou jovens dos 10 aos 18 anos quanto ao início dos sintomas depressivos e sua relação com a posição socioeconômica prévia da família, avaliada pela ocupação dos pais, anos de estudo da mãe e padrão de vida (dificuldades materiais, casa própria e acesso a carro). Houve associação entre depressão aos 18 anos e exposição a problemas financeiros e dificuldades materiais^10^. Uma avaliação da população geral a partir de 15 anos encontrou associação entre TMC e baixa renda e analfabetismo, entendendo que fatores socioeconômicos impactam a saúde mental em todas as fases da vida^17^. No Brasil, adolescentes com mães de menor escolaridade tiveram maior prevalência de TMC que os filhos de mães com mais de oito anos de estudo^7^^,^^8^.

Os estudos com adolescentes têm trabalhado mais com a escolaridade materna enquanto posição socioeconômica. Neste estudo, essa informação foi dada pelo próprio adolescente, em instrumento autopreenchido, e evidenciou-se incompletude dos dados referentes à escolaridade do chefe de família, condição que inviabilizou a classificação econômica. Nesse sentido, postula-se que possa ter havido um viés de informação quanto à escolaridade da mãe, e que o uso de outras variáveis *proxy* possa ter proporcionado uma maior compreensão das perguntas.

As variáveis utilizadas para avaliar a condição de vida e a socioeconômica neste estudo foram um *proxy*, como raça/cor, anos de estudo da mãe, número de moradores por cômodo, número de banheiros, presença de empregada doméstica mensalista, tipo de escola, localidade da escola e atividade ocupacional do jovem. Diante dos resultados, temos que questionar se essas medidas expressam a condição socioeconômica dos adolescentes.

Ewerling et al.^21^ avaliaram a posse de bens pelo indicador econômico nacional, também comum ao Critério Brasil da Associação Brasileira de Empresas de Pesquisa, ambos foram criados como um *proxy* que expressasse a riqueza das residências. O estudo trabalhou com dados de inquéritos bianuais realizados em Pelotas (RS) nos anos entre 2002 e 2014, e somente em 2012 havia adolescentes. Neste estudo, foram utilizadas de forma isolada três dessas variáveis: empregada mensalista, banheiro e relação morador/cômodo – marcadores de domicílios ricos^21^.

A avaliação da relação entre pobreza e saúde mental em países de baixa e média renda tem mostrado resultados distoantes, possivelmente pelo tipo de variáveis selecionadas para avaliar a condição socioeconômica, não alicerçadas por uma definição de pobreza. Em estudos com variáveis socioeconômicas como educação, renda e outras, é comum ver uma associação positiva na análise bivariada que enfraquece, desaparece ou fica negativa na análise ajustada^27^. Isso ocorreu também neste estudo, pois a escolaridade materna só mostrou diferença na análise bruta, que não se manteve na análise ajustada.

Os problemas de medição utilizando instrumentos de triagem como o GHQ, a maneira de medir a variável pobreza e os fatores populacionais podem explicar a variabilidade da associação encontrada nos estudos também pelos fatores de mediação da relação entre pobreza e TMC. Por outro lado, saber que a pobreza causa TMC não exclui o inverso, e estudos de coorte são necessários. Há dimensões específicas da pobreza que levam ao TMC, e os caminhos a serem percorridos são complexos, pois a posição socioeconômica é uma construção social e multidimensional^10^.

Como limitações do estudo, destaca-se que todos os instrumentos utilizados com crianças e adolescentes são passíveis de vieses de informação. O GHQ-12 é um instrumento amplamente validado em adultos, mas ainda carece de mais estudos em adolescentes. Todavia, encontraram-se estudos da análise fatorial do GHQ-12 aplicado em jovens brasileiros^14^ e australianos^16^, e um estudo longitudinal que avaliou a sensibilidade cultural do instrumento evidenciou que ele é culturalmente sensível para diversos grupos étnicos na Inglaterra^28^. Ainda há diferentes formas de pontuação, o que dificulta sobremaneira a comparação dos resultados com outros estudos. No entanto, ele tem sido o instrumento de escolha em estudos epidemiológicos sobre o tema, seja com adultos ou jovens.

As variáveis socioeconômicas que estiveram associadas positivamente a TMC nos adolescentes, como ter empregada e estudar em escola privada, são sugestivas de pertencimento a classes econômicas mais elevadas e sugerem que isso pode proporcionar um ambiente gerador de alterações do humor. O trabalho não remunerado favoreceu a saúde mental dos adolescentes e representa um tipo de atividade que limita a ociosidade e a exposição a ambientes de risco. Os resultados foram distintos da literatura sobre condição socioeconômica e TMC; recomenda-se a realização de outros estudos com adolescentes brasileiros para averiguar a validação externa.
